# The “wing-heeled” traveler

**DOI:** 10.1186/s40794-020-0103-y

**Published:** 2020-02-18

**Authors:** Taylor Kain, Jordan Weinstein, Aaron Thompson, Andrea K. Boggild

**Affiliations:** 1 Department of Medicine, University of Toronto, Toronto, Canada; 2 Division of Nephrology, St. Michael’s Hospital, Toronto, Canada; 3 Occupational and Environmental Medicine Clinic, St. Michael’s Hospital, Toronto, Canada; 4 Dalla Lana School of Public Health, University of Toronto, Toronto, Canada; 5 Tropical Disease Unit, Toronto General Hospital, 200 Elizabeth Street, 13EN-218, Toronto, ON M5G 2C4 Canada

**Keywords:** Ciguatera fish poisoning, Heavy metal intoxication, Nephropathy, Neuropathy, Skin bleaching

## Abstract

Intoxication syndromes may be travel acquired, and are related to intentional or accidental inhalational or percutaneous exposures or ingestions. Due to their myriad clinical presentations, initial differential diagnosis of such intoxications in returned travelers is broad, and typically requires detailed history and laboratory investigations to disentangle. We herein use a case-based clinical problem solving approach to illumination of a mercury intoxication syndrome, which presented in a 48-year-old VFR traveler to Guyana. Common clinical presentations, differential diagnoses, laboratory investigations, and therapeutic interventions are discussed.

## Background

Intoxication syndromes may be travel acquired, and are related to intentional or accidental inhalational or percutaneous exposures or ingestions. Examples include but are not limited to the marine intoxications - such as ciguatera, various shellfish poisonings (e.g., amnestic, neurotoxic, diarrhetic), *Sargassum* weed, and scombroid - as well as intoxication syndromes related to chemical exposures (e.g., organophosphates, anticholinergics) or heavy metals. Due to their myriad clinical presentations, initial differential diagnosis of such intoxications in returned travelers is broad, and typically requires detailed history and laboratory investigations along with close patient follow-up to disentangle.

We herein use a case-based clinical problem solving approach to illumination of a mercury intoxication syndrome, which presented in a 48-year-old VFR traveler to Guyana.

Using this case-based approach to the evaluation and diagnosis of a syndrome compatible with intoxication in the post-travel setting, the common clinical presentations, differential diagnoses, laboratory investigations, and therapeutic interventions are discussed.

## Case presentation

A 48-year-old woman was referred to our tropical disease clinic for malaise, fatigue, weakness, and paresthesia after a trip to Guyana. The patient was initially assessed at a peripheral hospital emergency department 1 month after her return from Guyana. She was then seen in an infectious diseases clinic and then referred to our clinic where she was seen about 2 months after her return.

The patient had been well prior to her trip to Guyana, where she spent 2 weeks visiting friends and relatives (VFR). About 1 week into her trip she reports many bites from what she believed were sandflies on the beach. After this she developed fevers and aches that resolved in 24 h. Several days after returning from her trip she reported body aches and pains, chills, sweats, and a sensation of fever, though none had been documented. She also noticed burning in her hands and feet as well as intolerance to heat. Around the same time, the patient developed some mild dysuria and vaginal itch and took an over-the-counter miconazole cream for yeast infection and was given nitrofurantoin by her family doctor. She complained of significant fatigue, diffuse weakness, and persistent paresthesia affecting her daily functioning.

Returning travelers often present to medical professionals with various health concerns. There are several important considerations raised by this patient and her travel to Guyana. Given the history of possible fever, malaria should be excluded as it is a medical emergency, and is common along the Western regions of the country. VFRs are much less likely to seek pre-travel advice or take malaria prophylaxis. Prompt exclusion of potentially life-threatening infections such as typhoid or other bacteremias should occur with blood cultures. The most likely explanation for her febrile illness is a non-specific viral syndrome, that is likely to self-resolve. Other travel-related considerations for this patient’s symptoms include post-infectious syndromes following infections with dengue, Zika, or other flaviviruses, and marine intoxications, such as ciguatera toxicity and scombroid. History should be gathered regarding her specific travel related exposures, including food and water consumption (specifically consumption of large reef fish), exposure to fresh water, exposure to mounds of rotting seaweed (e.g., *Sargassum* weed), other arthropod bites, and animal exposures.

The patient had not sought pre-travel advice, took no insect precautions, and ate local food. She received no pre-travel vaccinations. She ate no undercooked meats but did report eating riverine fish (Gilbaka, Basher, and Hassar), and as well as cooked crab. She used local water for cooking but drank only bottled water. She had no animal exposures but did swim in fresh water. There was no *Sargassum* accumulation on the beach. Her time was spent in a local village as well as the capital city. The patient had no medical history other than a prior prescription for a “water pill” for mild leg swelling over several month period, but this had been stopped in the past year. She was not taking any other regular medication. She had no known allergies. Social history was notable for birth in Guyana with immigration to Canada in 1973. Other than her recent visit to Guyana, her last trip was 16 years earlier. She was a non-smoker, non-drinker, and had never used any injection drugs. She had no tattoos or piercings and denied sexual contact for over 20 years. Due to her illness she was on sick leave from her job as a librarian.

Her history indicates consumption of several species of reef fish, which increases the possibility of ciguatera poisoning, particularly if those fish were > 2 kg. Evaluation of returning travelers necessitates consideration of non-travel related conditions as well, and in this case, would include rheumatologic disorders such as rheumatoid arthritis, inflammatory or drug-induced myositis, sarcoidosis, and polymyalgia rheumatica; neurological disorders such as early multiple sclerosis with primarily sensory deficits; non-marine intoxications including heavy metal poisoning, botulism, and organophosphate toxicity; and endocrinologic or metabolic disorders such as hypothyroidism or vitamin B12 deficiency. Her symptoms could also be caused by an intoxication related or unrelated to her travel; as such, a detailed exposure history should be gathered. Sexual history should be obtained for risk factors of acute or chronic HIV infection.

When the patient was evaluated in our clinic, she was in no acute distress, but her weakness had progressed to the point of being wheelchair bound and requiring a two-person assist for transfer. She was afebrile, heart rate was 90 bpm, and blood pressure was 102/80 mmHg. There was no evidence of lymphadenopathy, oral ulcers, or pharyngeal erythema. Thyroid was unenlarged and of normal consistency without palpable nodules. Skin examination revealed no rashes or lesions. Cardiac and respiratory examinations were unremarkable, with no peripheral edema, wheezes, or other adventitious sounds. Abdominal examination was unremarkable and without evidence of hepatosplenomegaly. Neurological examination revealed normal cranial nerves, and normal sensation to light touch throughout all dermatomes. Pain sensation was normal throughout. Power was diminished throughout at 3 to 4- out of 5 in all muscles groups with no specific pattern and no proximal weakness noted. Deep tendon reflexes were difficult to elicit, but intact at the knees and ankles. There were no fasciculations notes, and tone was normal in all extremities. There were no other focal neurological deficits noted.

Based on symptoms and possible epidemiological risks, ciguatera toxicity is suspected, though post-viral syndrome is most likely. She does not describe the classic cold-hot reversal specific for ciguatera toxicity, nor does she endorse having eaten predatory reef fish known to be associated with ciguatera poisoning, but does report prominent burning sensation and paresthesia in the context of fish consumption abroad. Although she recalls only eating riverine fish, the possibility of a mixed or improperly labelled fish dish exists, particularly in the context of the current global fisheries. This patient should be counselled to avoid fish, nuts, caffeine, alcohol, and excessive exercise, all of which contribute to symptoms of ciguatera toxicity. Further work-up should be done to rule out competing causes as there is no confirmatory test for ciguatera toxicity, and should target other potential infectious causes, rheumatological or muscle disease, vitamin deficiencies, and renal disease. Basic investigations should include CBC, electrolytes, creatinine/BUN, and liver enzymes. Hepatitis A and B, HIV, a basic rheumatologic panel (ESR/CRP, CK, ANA, rheumatoid factor), and vitamin B12 levels should be sent. Urinalysis should be obtained given the ankle swelling and potential for an inflammatory condition. Given the history of fever equivalents (chills and sweats), infectious work-up should be completed with blood cultures; malaria thick and thin smears with rapid diagnostic test; and serologies for chikungunya, dengue, Zika and *Brucella*. Routine age-appropriate preventative health, including a mammogram and a pap smear, is also advisable.

The patient was seen in follow-up approximately 3 weeks after initial clinic visit. At this visit, she noted slight improvement in her symptoms with cessation of ciguatera dietary triggers, but there was ongoing burning muscle pain and sweats without fevers. Blood work revealed anemia (hemoglobin 8.9 g/dL), and thrombocytosis (platelets 649,000 /μL). White blood cell count (WBC) and differential were normal (WBC 7.6 × 10^3^/ μL). Electrolytes were within normal range, and creatinine was 0.92 mg/dL. ESR was elevated (28 mm/hr), but CRP was undetectable (< 3 mg/dL). Liver enzymes and bilirubin were within normal range. Infectious tests revealed negative malaria thick and thin smears, negative blood and urine cultures, and non-reactive IgG and IgM for chikungunya. Dengue testing showed a non-reactive IgG and an indeterminate IgM. CMV IgM had low reactivity while IgG was negative. Chest x-ray was normal. *Brucella* serology was negative. ANA and rheumatoid factor were negative. Calcium was 9.18 mg/dL (normal 8.5–10.5 mg/dL) and CK was 28 IU/L (normal 20–160 IU/L). SPEP and UPEP were negative. Urinalysis was notable for proteinuria, quantified at 1 g/L, with only trace blood, and no leukocytes/nitrites. There were no casts reported on urine microscopy.

Given the chronicity and multiplicity of symptoms, some of which improved with ciguatera trigger cessation, the multiple negative investigations, and non-specific reactive serologies, ciguatera or post-viral syndrome remain top differential diagnoses. The indeterminate dengue IgM is likely non-contributory in the context of non-reactive IgG as over a month since returning from her travels had elapsed. The normal CK mostly excludes an inflammatory myositis, and the negativity of her remaining rheumatologic panel makes other inflammatory diseases unlikely. The moderate proteinuria is significant and should be followed up with a 24-h urine collection to further quantify. The absence of leukocytes or casts and minimal blood make glomerulonephritis or other renal disease less likely.

At her initial visit, heavy metals were ordered due to the history suggestive of neuropathy, but due to performance at a regional reference laboratory, results were unavailable until her third follow-up visit. Blood mercury level was 35 μg/L which was above the reported reference range but below the critical alert value of 50 μg/L. She was called and informed of the result and referred to occupational health for assessment. At her occupational health assessment, careful exposure history was obtained through the use of a standardized questionnaire used to identify potential sources of mercury exposure. The only identified sources of mercury exposure were methylmercury from consumption of fish she had eaten in Guyana and elemental mercury from a single dental amalgam.

Blood mercury measurements reflect all types of mercury exposures; organic, elemental, and inorganic. Acute methylmecury toxicity tends to present with central nervous system dysfunction with a predilection to the visual and somatosensory cortices and cerebellum, manifesting as perioral paresthesia, visual disturbances and ataxia. The patient did not present with these symptoms and, additionally, the patient’s blood mercury levels, while elevated, were below levels typically associated with overt symptoms. Thus, organic mercury poisoning from fish consumption seems unlikely as the etiology for her symptoms.

The urine is the primary route of elimination for inorganic mercury while only a small fraction of organomercury compounds are renally excreted. As such, urine mercury levels are useful in assessing inorganic mercury exposure A 24-h urine mercury level should be ordered to exclude inorganic mercury toxicity.

Her proteinuria motivated an abdominal ultrasound, which was notable for mild pelviectasis bilaterally, with normal size and echogenicity of kidneys, and no evidence of hydronephrosis or stones. It also prompted referral to nephrology where she was followed in clinic due to increasing proteinuria over the next three months. Approximately six months after she returned from Guyana, proteinuria had progressed to 4.03 g/L and a 24-h urine collection was revealed 6.93 g protein/24-h. By this point, urinalysis also showed 3+ blood as well as leukocytes. Serum albumin was 33 g/L, and lipids were elevated with trigylcerides of 5.23 mmol/L, total cholesterol of 7.54 mmol/L, and non-HDL cholesterol of 5.9 mmol/L. Creatinine remained normal at 0.92 mg/dL. Repeat physical exam now revealed 1+ pitting edema in the legs bilaterally.

This patient now has nephrotic range proteinuria, with albumin on the low end of normal, and mild edema; nephrotic syndrome is a diagnostic consideration but not all criteria are present. There are reports of nephrotic-range proteinuria with mercury toxicity, but other considerations for her proteinuria include both non-proliferative etiologies of glomerulonephritis (GN) and, less likely, proliferative ones. Non-proliferative etiologies include: diabetic nephropathy; membranous nephropathy; focal segmental glomerulonephritis; minimal change nephropathy; and amyloidosis. These disorders can occur idiopathically or secondary to known associated conditions. Proliferative conditions include: IgA nephropathy; SLE; post-infectious GN; cryoglobulinemia; and much less likely anti-glomerular basement membrane disease or vasculitis. Inherited disorders such as Alport Syndrome or Fabry disease would be less common. Other uncommon causes of GN which nevertheless could be considered are MPGN and non-amyloid fibrillary GN.

As her proteinuria continued to worsen, renal biopsy was pursued, which showed membranous glomerulonephritis. Staining for PLA2R was negative; immunofixation for IgM, IgA, C3, and C1q were not significant.

Membranous nephropathy (MN) is characterized by diffuse basement membrane thickening without significant hypercellularity. On electron microscopy, dense deposits appear in the sup-epithelial space interspersed with basement membrane, producing the spikes characteristic of this disorder. Excluding diabetes, MN is the most common cause of idiopathic nephrotic syndrome in non-black adults. MN can be idiopathic or secondary to another disease process. These include but are not limited to infections (viral hepatitis), SLE, toxins and drugs (gold, penicillamine, NSAIDs), and solid tumors.

Repeat blood mercury level performed 2 months after initial mercury level was obtained was now 88 μg/L, which was well above the action level. Urine mercury level was also extremely elevated at 448 μg/L (reference range 0–3 μg/L). The patient’s mother (with whom she lived) was also tested for blood and urine mercury levels to exclude a shared source of home exposure. She had a normal blood mercury level, but urine mercury level was elevated at 10.5 μg/L. This suggests a shared inorganic mercury exposure source with the patient having a higher degree of exposure than her mother. The exposure history was repeated with the patient’s mother. The patient reported that she had been using a skin lightening cream consistently for the past 3 years. This was the same cream as her mother was using and, indeed, she reported using it on a daily basis in similar amounts to what one would use for a moisturizer. A sample of the cream was sent to the provincial public health laboratory for testing, which revealed an inorganic mercury level of over 13,000 mcg/g.

A diagnosis of inorganic mercury poisoning was made on the basis of laboratory investigations demonstrating elevated urine mercury levels with a confirmed source of exposure (skin lightening cream). The mainstay of treatment is removal from exposure. Chelation therapy was considered; however, based on the current literature it remains unclear whether therapy with chelating agents is truly beneficial for mercury intoxication and indications for therapy have not been established.

The patient was instructed to stop using the skin lightening cream. Serial blood and urine mercury levels were monitored and showed decreasing levels consistent with the half-life of inorganic mercury in the body. The weakness and subjective paresthesia began to resolve, and she regained full strength within several months of cessation. Her nephrotic-range proteinuria began to improve as her serum mercury levels fell, with the most recent 24-h urine collection demonstrating 150 mg of protein, which is within in the normal range.

### Commentary

Ciguatera is a marine toxicity that originates in the plankton species of genus *Gambierdiscus* on coral reefs [1, 2]. It is spread to humans after eating large reef fish, such as snapper, barracuda, grouper, and eels. It is not destroyed by cooking/freezing, and so food handling is not implicated in ciguatera toxicity. Hot-cold reversal is nearly pathognomonic for ciguatera toxicity but is present in only 50% of cases. Other symptoms may be neurological: paresthesia (especially peri-oral), ataxia, and headaches; gastrointestinal: nausea/vomiting, diarrhea, and abdominal cramping; or cardiac: bradycardia, heart blocks, and hypotension. Myalgia, pruritus with rash, fatigue, and dysuria are also common [3].

While there were some features consistent with ciguatera toxicity, one of the top presumptive diagnoses along with post-viral syndrome during her initial work-up and treatment, we do not believe that ciguatera toxicity accounted for her clinical presentation, laboratory derangements, and glomerulonephritis. The fish she ate were not classic culprits, and her symptoms were atypical. Rather than ciguatera toxicity, her symptom constellation and entire clinical picture most likely are attributable to underlying mercury toxicity, which triggered an autoimmune GN. The initial subjective fevers were either a red herring, or reflective of a non-specific, self-limited viral infection.

Mercury in its three forms – elemental, inorganic, and organic – may cause toxicity [4–8] (Table 1). Toxicity differs by type and acuity of exposure. The most common forms of mercury to which the general population is exposed are methylmercury from dietary consumption of fish and elemental mercury from dental amalgams.

**Table 1:** Summary of common forms of mercury, their sources of exposure, major symptoms, and method of excretion with biological half-life

Form	Source(s)	Absorption	Target Organ(s)	Signs/Symptoms	Major Excretory Pathway	Approximate Biological Half-Life
Elemental (Hg^0^)	Occupational Dental Amalgam	Inhalation (vapour)	CNS, PNS, Kidney	Insomnia, anxiety, tremor, peripheral neuropathy, nephropathy	Urine and Feces	60 days
Inorganic Hg Salts	Topical antiseptics, cosmetics through disrupted skin/epithelium	Ingestion, dermal	Gut, Kidney	Nausea, vomiting nephropathy	Urine and Feces	40 days (typical range 1–3 months)
Methyl Hg	Diet (fish)	Ingestion	CNS	Perioral paresthesia, constriction of visual fields, dysarthria, ataxia, impaired hearing, tremor	Feces	40–70 days
Ethyl Hg	Thimerosal containing vaccines	Parenteral	CNS, Kidney	Ataxia, paraparesis, deafness, constricted visual fields	Feces	7–20 days

* Adapted from Clarkson TW, Magos L, Myers GJ. The toxicology of mercury – current exposures and clinical manifestations. *N. Engl. J. Med.* 2003;349:1731–7

Methylmercury has a predilection for the central nervous system. Significant outbreaks of methylmerucy toxicity have occurred from consumption of contaminated grains and consumption of fish from mercury polluted waters yielding dose-response relationships for severe acute toxicity. Symptoms primarily reflect central nervous system effects and include paresthesia (especially perioral), malaise, visual field defects, tremors, peripheral neuropathy, ataxia, and neuropsychiatric symptoms. At lower doses, methylmercury is toxic to the developing fetal nervous system and current reference doses are primarily set based on endpoints reported in studies considering in utero exposure sequelae in the fetus. Research on chronic low level methylmercury exposure is evolving and suggestive of potential neurocognitive decrements though dose-response curves and potential effect modifiers remain poorly defined [9].

Sources of elemental mercury exposure include dental fillings, vapors from broken thermometers, compact fluorescent light bulbs, and occupational exposures such as gold mining. Inhalation of mercury vapors can result in pulmonary, neurological, and nephrotoxicity. Inorganic mercury is a salt that can be ingested or absorbed from the skin. Sources include ayurvedic and other traditional medicines, hair dyes, and skin-whitening creams. Acute ingestion can result in hemorrhagic gastroenteritis, cardiovascular collapse and acute tubular necrosis. Chronic exposure can cause mood changes, tremor, colitis, excess salivation, and nephrotic syndrome. Nephrotic syndrome is rare complication of mercury toxicity. The underlying renal pathology in most reported cases is membranous nephropathy which is attributed to an idiosyncratic hypersensitivity reaction [10, 11]. The mainstay of treatment is identification and elimination of the exposure source, followed by serial biomonitoring. Steady decline in urinary mercury levels in keeping with the typical biological half-life, which may vary between 1 and 3 months, confirms the source has been eliminated [12]. The role of chelation therapy remains controversial for mercury toxicity and there are currently no established indications for therapy [13].

Cosmetics are well established sources of both organic mercury, which is used as a cosmetic preservative, and inorganic mercury which is commonly used as a skin lightening agent [14]. Skin bleaching creams are used in many countries worldwide, particularly in Sub-Saharan Africa, the Middle East, Asia, and Central and South America [15, 16], and inorganic mercury has been documented in such creams even in North America [17]. Whitening creams are often applied once to twice per day and used for decades due to the rapid reversibility of skin lightening upon product cessation [18]. Recently, three children in Sydney Australia became ill after cutaneous exposure to a popular Kohl-based eyeliner containing 84% lead, as well as toxic levels of cadmium, mercury, and arsenic, thus highlighting the ongoing public health threat posed by such products [19].

The most common side effects associated with skin lightening creams are skin disorders including superficial mycoses, scabies, superficial bacterial pyoderma, cellulitis, steroid-induced acne, and striae. Glomerulonephritis and neurological complications are described [20], and corroborated by our case. Migrants from areas where skin bleaching practices are common often import such behaviors to communities in other countries, therefore practitioners should be aware of potential adverse outcomes associated with their use, regardless of practice location. Obtaining a definitive endorsement of skin bleaching on history is difficult, and to preserve the therapeutic alliance, it is likely best to avoid condemnation of the practice. Neutral language explaining the risks associated with bleaching, especially during pregnancy, is preferred with such patients. Strong social pressures may exist from peers, communities, and advertising, and lighter skin is often viewed as a sign of higher socioeconomic level [21].

We determined that this patient had been using her skin whitening cream for several years without disclosing to health care professionals. Even with careful probing she was only partially forthcoming, and where she had obtained the cream was unclear, though we suspect that she had purchased a recent supply in Guyana. The mildly elevated blood mercury level initially does not rule out inorganic mercury poisoning. Since inorganic mercury is primarily cleared through the kidneys, blood levels can be only mildly elevated despite toxic side effects. This scenario is likely what was occurring in this case.

## Conclusions

Mercury remains an important environmental risk both internationally and in migrant communities, and as such clinicians should be vigilant for potential exposures in their patients, especially as patients may not openly provide history suggesting exposure. Due to the myriad presentations of intoxication syndromes that might be encountered in a post-travel setting, detailed history and physical examination, comprehensive laboratory testing, and close patient follow-up are required. The differential diagnosis of intoxication syndromes in the post-travel setting may include the aforementioned infectious diseases, along with ingested marine poisons (such as those of the various shellfish poisonings), chemical inhalational exposures (such as hydrogen sulphide of *Sargassum* weed or organophosphate poisonings), or percutaneous exposures from recent or chronic application of topical agents, as was seen in this case.

## Data Availability

All data are presented herein.
